# Laparoscopic Approach To Treat a Hydrocele of the Canal of Nuck: A Case Report

**DOI:** 10.7759/cureus.68475

**Published:** 2024-09-02

**Authors:** Omer K Alghofeali, Wed K Alwabel, Mohammed A Alharbi, Ghassan I Alhajress, Ibrahim T Al Babtain

**Affiliations:** 1 Department of Medicine, King Saud Bin Abdulaziz University for Health Sciences College of Medicine, Riyadh, SAU; 2 Department of Pathology and Laboratory Medicine, King Abdulaziz Medical City Riyadh, Riyadh, SAU; 3 Department of Urology, King Abdulaziz Medical City Riyadh, Riyadh, SAU; 4 Department of General Surgery, King Abdulaziz Medical City Riyadh, Riyadh, SAU

**Keywords:** hydrocele of nuck, biosynthetic mesh, mesh repair, inguinal hernia mesh repair, laparoscopic hernia repair, canal of nuck hydrocele, absorbable mesh, laparoscopic treatment, the canal of nuck, hydrocele of the canal of nuck

## Abstract

The hydrocele of the canal of Nuck is a rare medical condition that usually affects females during childhood and early adulthood. It is considered the female homolog to the testicular hydrocele in males, as they share similar pathophysiology.

The condition is often underreported and considered an incidental finding. On many occasions, it is mistakenly diagnosed and even managed as an inguinal hernia. The hydrocele of the canal of Nuck is usually managed surgically, either by open surgery or laparoscopy.

In this case report, we will discuss the hydrocele of the canal of Nuck diagnosed in a young adult female and provide a background, case presentation, and thorough discussion.

## Introduction

The hydrocele of the canal of Nuck (HCN) is a rare and underreported condition that affects young adult females. During the normal development of a female fetus, the processus vaginalis, a pouch of the parietal peritoneum that continues through the inguinal canal along with the round ligaments, is normally obliterated early in life. However, in rare cases, this obliteration may fail, giving rise to the canal of Nuck that can develop into a hydrocele. The usual presentation is swelling in the inguinal area, which is why this condition is very commonly misdiagnosed as an inguinal hernia. When the possibility of the presence of a HCN arises, a diagnosis can be reached by relying on multiple radiological modalities, including ultrasound and CT scan, which we used for this case. The main and most effective method of treatment is the complete excision of the HCN, either by open surgery or laparoscopy, followed by hernioplasty [1]. In addition to surgery, other treatment methods have been described in the literature, including sclerotherapy or aspiration [2]. In this case report, we discuss the case of a young female who had a HCN excised and repaired laparoscopically using a bio-synthetic absorbable mesh.

## Case presentation

**Figure 1:**
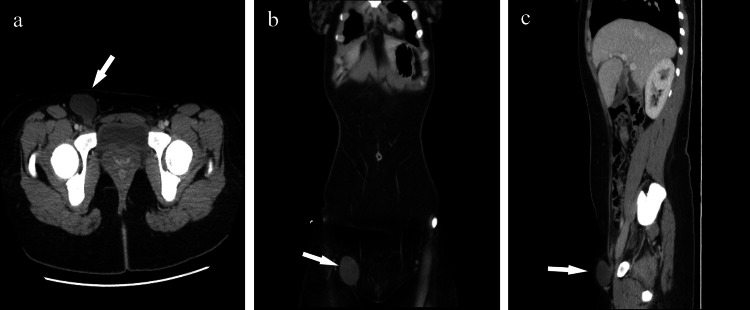
CT scan of the abdomen and pelvis showing the location of the hydrocele. Three CT scan images depict the hydrocele of the canal of Nuck (white arrows) in the right groin through (a) horizontal, (b) coronal, and (c) sagittal sections.

A 20-year-old female, not known to have any medical illnesses and with an unremarkable surgical history, presented to the general surgery outpatient clinic at the main hospital of King Abdulaziz Medical City (KAMC) in Riyadh, Saudi Arabia. She complained of right inguinal swelling. On physical exam, the patient felt uncomfortable but not in pain. She had a unilateral inguinal swelling on the right side that was tender and non-reducible with a mild cough impulse. The rest of the examination was unremarkable. An enhanced CT scan of the abdomen and pelvis with IV contrast was ordered and later revealed a thin-walled, uniloculated, and fluid-filled cyst measuring 3.8*3.3 cm. These radiological findings are consistent with the HCN (Figures 1a-1c).

**Figure 2:**
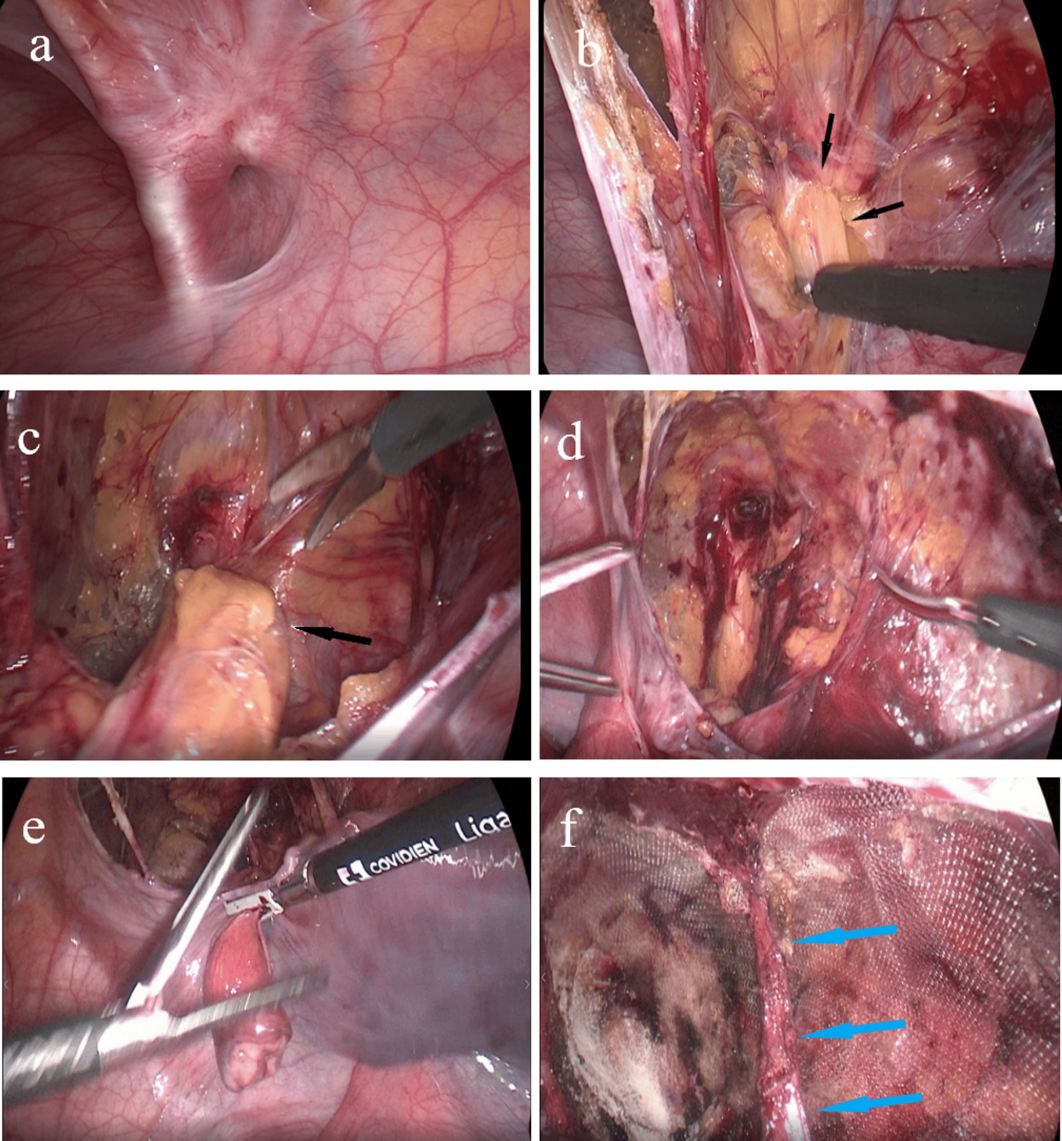
Laparoscopic procedure to remove the hydrocele and repair the defect. These six intraoperative pictures show the following: (a) the abdominal wall and the defect before excision; from (b) to (e) the process of dissecting and then removing the hydrocele (black arrows); (f) the repair of the defect using a 10*15 cm bio-synthetic slow absorbable mesh placed behind the round ligament (blue arrows).

The patient was admitted for elective day-time surgery. A laparoscopic right inguinal cyst excision followed by the application of a bio-synthetic slow absorbable mesh to seal the defect caused by the cyst was performed. During surgery, we localized and then reduced the hernia sac and its content completely, preserving the round ligament. The content was a fluid-filled cyst with a small fat content (Figures 2a-2e). The specimen was removed successfully using an endobag to preserve it. Then, we applied a 10*15 size bio-synthetic slow absorbable mesh just behind the round ligament, which was fixed using glue all around (Figure 2f). The specimen was placed in one container of formalin and sent to the Department of Pathology.

**Figure 3:**
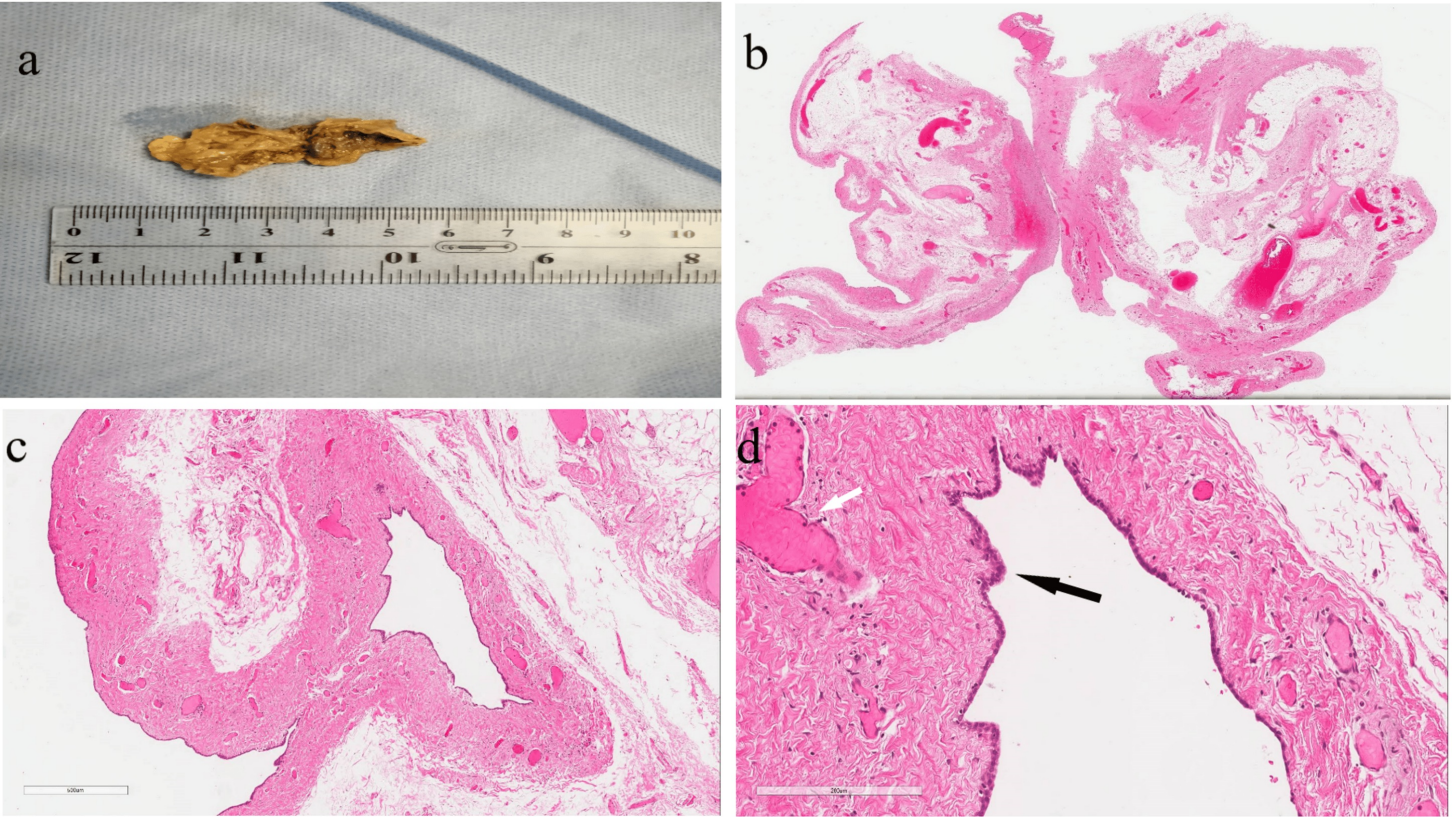
Pathology of the hydrocele of Nuck. (a) This picture shows the outer and internal structures of the specimen, with noticeable fatty tissue surrounding the cyst. Multiple micrographs show the histological structures of the hydrocele of Nuck at different magnifications: (b) 10x, (c) 100x, and (d) 200x. (b) Signs of inflammation, hemorrhage, and blood vessel congestion are visible across the picture. (d) Notice the congested blood vessel (white arrow) and the mesothelial lining (black arrow) depicted here.

The specimen was received at the Department of Pathology. During the gross examination, the specimen consisted of a unilocular cyst surrounded by yellow-tan fibrofatty tissue measuring 6.0*4.0*1.5 cm. The cyst's outer surface was gray-tan and smooth, and the inner surface was gray-brown with a wall thickness measuring up to 0.1 cm (Figure 3a). Representative sections were submitted in three cassettes. During microscopic examination, the section showed a cyst lined by benign low cuboidal to columnar epithelial cells (Figure 3d), and surrounded by fibro-fatty tissue with chronic granulomatous inflammation and congested blood vessels (Figures 3b-3d).

## Discussion

The canal of Nuck, which is the homolog to the male's patent processus vaginalis in the female inguinal canal, was initially documented by Dutch anatomist Anton Nuck. A failure to obliterate the distal segment of the canal will result in the formation of a fluid-containing sac known as the HCN [3]. The inguinal canal is a 4 cm long channel in the lower abdominal wall that connects tissues from the peritoneum to the perineum. The embryology of the inguinal canal is linked to two structures: the gubernaculum and the processus vaginalis [4]. The gubernaculum is a fibromuscular cord that develops in the fetus between the ages of eight to 12 weeks [5]. It joins the midsection of the uterus in females. Above this connection, it becomes the ovarian suspensory ligament, which holds the ovarian arteries and prevents the ovary from descending into the inguinal canal. It forms the round ligament that goes into the inguinal canal and attaches to the labia majora inferior to the uterine connection. Females have a shorter processus vaginalis, which is part of the parietal peritoneum that invaginates and descends anteriorly to the gubernaculum [4,5]. The superior section of the processus vaginalis typically merges at or around birth, with caudal obliteration continuing until the entire structure shuts within the first year of life [6]. When the processus vaginalis fails to obliterate completely or partially, a potential gap known as the canal of Nuck forms. An HCN might present clinically as a painless or painful fluctuant inguinal mass with no nausea or vomiting. According to some existing literature [7-9], the mass can be manually reduced and, when performing the Valsalva maneuver, it doesn’t show any increase in volume. The inguinal hernia, which is frequently confused for HCN, is the most essential differential diagnosis [10]. The best initial way to diagnose an HCN is by ultrasound. A well-defined anechoic lesion with posterior acoustic amplification often characterizes HCN on ultrasonography. Furthermore, MRI is used to have a greater grasp of the herniated structures. On T1-weighted imaging, HCN is frequently hypointense, however, on T2-weighted imaging, it is hyperintense. An MRI can help in differentiating the HCN from other soft tissue tumors that could appear as a palpable inguinal mass [11]. Usually, the diagnoses of the HCN using CT are incidental findings due to its usage for nonspecific abdominal pain at the emergency department.

According to the literature, patients' presentations might differ. HCN may present as painful or non-painful swelling in the inguinal area and labia majora. Our patient was a 20-year-old female who presented with right-sided inguinal swelling that was not painful. Similarly, Wang et al.'s case involved a 28-year-old female presented with swelling that was not painful in the right groin and was suspected to be an inguinal hernia. On the other hand, Aldhafeeri et al. and Shahd et al.'s patients were 25- and 36-year-old females respectively who presented with painful right-sided swelling. In terms of diagnosis and investigation, our CT scan of the abdomen and pelvis revealed a thin-walled uniloculated, and fluid-filled cyst measuring 3.8*3.3 cm. Likewise, Aldhafeeri et al.'s CT of the abdomen showed similar features. However, the diagnosis of our case was confirmed intraoperatively since HCN is rare we kept other differentials. Asymptomatic effusion or hernia caused by an unclosed HCN can result in the protrusion of abdominal organs, usually the intestines and ovaries which can result in life-threatening conditions such as strangulation, intestinal obstruction, or ovarian torsion [12]. Because of these possible complications, rapid identification and treatment of HCN are crucial.

## Conclusions

In this case report, we showed how the HCN can be effectively diagnosed using a CT scan with IV contrast, even though HCN is rare and commonly misdiagnosed. The patient underwent successful surgery to remove the hydrocele and repair the defect with slow absorbable bio-synthetic mesh, with no intraoperative or postoperative complications. This case report will provide useful data for clinicians who may encounter this rare condition and will offer them helpful information in the future to develop an accurate diagnosis and effective management plan for the HCN.
